# Accurate Prediction of y Ions in Beam-Type Collision-Induced Dissociation Using Deep Learning

**DOI:** 10.1021/acs.analchem.1c03184

**Published:** 2022-05-24

**Authors:** HyeonSeok Shin, Youngmin Park, Kyunggeun Ahn, Sungsoo Kim

**Affiliations:** Bio Convergence Research Institute, Bertis Inc., Heungdeok 1-ro, Giheung-gu, Yongin-si, 16954 Gyeonggi-do, Republic of Korea

## Abstract

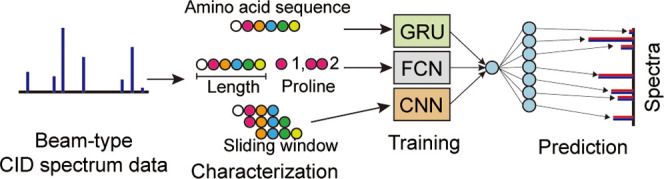

Peptide fragmentation spectra contain critical information for the identification of peptides by mass spectrometry. In this study, we developed an algorithm that more accurately predicts the high-intensity peaks among the peptide spectra. The training data are composed of 180,833 peptides from the National Institute of Standards and Technology and Proteomics Identification database, which were fragmented by either quadrupole time-of-flight or triple-quadrupole collision-induced dissociation methods. Exploratory analysis of the peptide fragmentation pattern was focused on the highest intensity peaks that showed proline, peptide length, and a sliding window of four amino acid combination that can be exploited as key features. The amino acid sequence of each peptide and each of the key features were allocated to different layers of the model, where recurrent neural network, convolutional neural network, and fully connected neural network were used. The trained model, PrAI-frag, accurately predicts the fragmentation spectra compared to previous machine learning-based prediction algorithms. The model excels at high-intensity peak prediction, which is advantageous to selective/multiple reaction monitoring application. PrAI-frag is provided via a Web server which can be used for peptides of length 6–15.

Proteomics is a growing field of research that has greatly benefited from the advances in tandem mass spectrometry (LC–MS/MS) technology. Increased throughput, accuracy, and resolution of MS/MS have enabled the rapid transition from peptide discovery to industrial and clinical applications.^1^ One of the breakthroughs in MS/MS technology lies in the process of peptide fragmentation and isolation of its fragments. Historically used collision-induced dissociation (CID) with ion trap CID (IT-CID), also known as resonance-type CID, uses relatively low energy for fragmentation. Beam-type CID, a fragmentation method that uses relatively higher collision energy (CE), such as triple quadrupole (QqQ), quadrupole time of flight (QTOF), and higher energy collision dissociation (HCD) were developed later.^2−4^ Among the different fragmentation methods, QqQ, QTOF, and HCD methods have gained attention as higher CE increased the detection of y ions which enabled the detection and quantification of low-abundant proteins.^2,3,5,6^

The differences in fragmentation methods, instruments, number of collisions, and CE strength lead to different patterns of fragmentation. The IT-CID method leads to different fragmentation patterns compared to beam-type CIDs at high CE settings, whereas similar spectra are obtained on QqQ and QTOF.^3^ To identify peptides from different fragmentation methods, database generation for each fragmentation method would be required. Unless sufficient databases have been made, the database search method may result in a large fraction of unidentified spectra, which led to increased development of more powerful prediction algorithms.^7,8^ In particular, HCD method has recently been widely used for scanning complex data in a high-throughput manner, leading to an explosive increase in available data. Along with the subtle but unignorably different fragmentation pattern of HCD compared to other types of CID, deep learning has recently been employed in several studies to predict the fragmentation spectra.

Deep learning and machine learning have been utilized to predict fragment patterns in several studies.^9,10^ As peptides are composed of amino acids, the amino acid combination is approached in a similar manner to the time series or natural language data, which is highly applicable for recurrent neural network (RNN) models.^11−14^ Furthermore, sequence data are converted into image or vector data with additional information, as features have been utilized for applications to convolutional neural networks and existing machine learning algorithms.^12,15^

Regardless of the trend in the research community, clinical industries are showing more interest toward selective/multiple reaction monitoring (SRM/MRM) MS/MS technology which uses QqQ CID as the fragmentation method.^16^ The relatively lower resolution of MRM is compensated by its reproducibility and the ability to quantitate multiple biomarkers in a single run, which makes MRM a favorable instrument for clinical application. To this end, we developed a deep learning algorithm that is more suitable for such applications. The model is composed of RNN, convolutional neural network (CNN), and fully connected neural network (FCN), each allocated to appropriate features. The algorithm was more specifically fitted to QTOF CID fragmentation and trained to focus on the prediction of y ions (peptide fragments on the C-terminus) that are expected to be measured at higher intensities by MS/MS.^17^

## Methods

### Data Preparation

The data used for training in this study are composed of the National Institute of Standards and Technology (NIST) collision cell library quadrupole time-of-flight of human data (2012-04-20), NIST yeast data (2012-04-17), data from the Proteomics Identification (PRIDE) study of human proteins by SWATH-MS (PXD000954), and 647 laboratory-synthesized peptides that are part of Pep-Quant library (Bertis).^18,19^ The NIST rat data (2013-06-05) were used for overfitting validation and model evaluation.^18^ Additional evaluation of the models was performed on the PRIDE data PXD001587 and PXD008651.^20,21^ All obtained data were first parsed to the NIST database format. For peptides that are found in more than one database, a single database with the highest number of peaks in the spectrum was used. Modifications, such as carbamidomethylation on cysteine, were ignored and encoded as unmodified sequences. Peptides with the same sequence but different charged states were considered unique peptides. The amino acid sequence of each peptide was one-hot encoded to 20 numbers, each representing an amino acid. The b ions were removed. The peak intensities for a peptide were transformed to a size of (1,42) tensor, where each y ion was allocated three times to annotate the charge state of 1 to 3, from Y1 to Y14. The missing values that were highly unlikely or impossible to exist, such as Y14 ion for peptide length 8, were filled with −1. Otherwise, the missing values were filled with 0.

### Training Environment

The deep learning model was generated using Python ver. 3.8.3 environment with Python libraries, Torch ver. 1.7.1,^22^ numpy, 1.18.5,^23^ pandas ver. 1.2.2, pyteomics ver.4.3.2,^24^ sklearn ver.0.0,^25^ pyYAML ver. 5.4.1, and easydict ver. 1.9. Unless otherwise mentioned, the training was performed via PyTorch model pipelines with MSEloss (mean squared error loss, reduction = “mean”), Adam optimizer (LR = 0.01),^26^ ReduceLROnPlateau scheduler of mode “min”, gamma value of 0.1, and patience of 7. The training was performed using GeForce RTX 3090 (NVIDIA) with CUDA ver. 11.2.

### Training Model Structure

The training model initially used the peptide amino acid sequence, charge, and CE as input values (Supporting Information Data S1). Using the input values, the length of the peptide and the counted number of proline residues in the peptide were calculated to be added as an extra feature in the model. In addition, the sliding window of the 4-mers were generated as vectors and added as another feature. The structure of the training model is a combination of RNN, FCN, and CNN with conventional layers as follows; the one-hot encoded sequence was weighted by a GRU bidirectional layer with a hidden size of 128. Part of the hidden information was stored in memory, and part of the information was passed through dot product attention layers that focus on the important features obtained from GRU, which are merged to the products of the FCN and CNN. The information from CE, charge, peptide length, and proline residue count were inputted through a FCN layer. The initial linear vector form of the sliding window of 4-mers is transformed to a 2D matrix of 4 × 12. The transformed 2D data were convoluted through three CNN layers. The results from the FCN and CNN are concatenated first and merged to the results from the first GRU layer by matrix multiplication, which was decoded by the second GRU layer. The weights were then processed into a second dot product attention layer and linear layers that form the prediction (Supporting Information Data S2).

### K-Fold Validation

To validate the model’s accuracy and check for overfitting, the training data were divided to 10-fold, where 1 out of 10 was used for validation and 9 out of 10 were used for training. During k-fold cross-validation training and validation, the data change for each fold, enabling each data to be used as the validation data at least once. Each fold generated its final model after training, which was evaluated by the Pearson correlation coefficient (PCC) calculation on the NIST rat data.

### Model Comparison

Prosit_2020_intensity_hcd, MS^2^PIP_QTOF and MS^2^PIP_HCD were used in comparison with our model (Supporting Information Data S3). For our model, from the 10 models obtained from k-fold cross-validation, the fold 2 model was used in PCC comparison, highest peak accuracy comparison, and in the inference model provided by the Web site. PCC was calculated using two methods. The first method, which will be called “without-zero” throughout the manuscript, uses input values that exist in the database (Supporting Information Figure S1a,b). For example, for a peptide “A” of length 9 and charge 2, the spectrum information for Y8, Y7, Y6, and Y5 is in the database. Then, the predicted values for Y8, Y7, Y6, and Y5 are compared. The second method, which will be called “with-zero” throughout the manuscript, uses input values with zero filled in for fragments without detected intensity. For peptide “A”, the intensity prediction was compared for Y8^+2^, Y8, Y7^+2^, Y7, Y6^+2^, Y6, 25^+2^, Y5, Y4^+2^, Y4, Y3^+2^, Y3, Y2^+2^, Y2, and Y1.

### Inference

The inference model is available at www.prai.co.kr and https://github.com/bertis-prai/prai-test. The only required inputs are the peptide sequences, and the enabled inputs are charge and CE value, which are filled in if missing (Supporting Information Data S1).

## Results and Discussion

### Input Data Preparation

**Figure 1:**
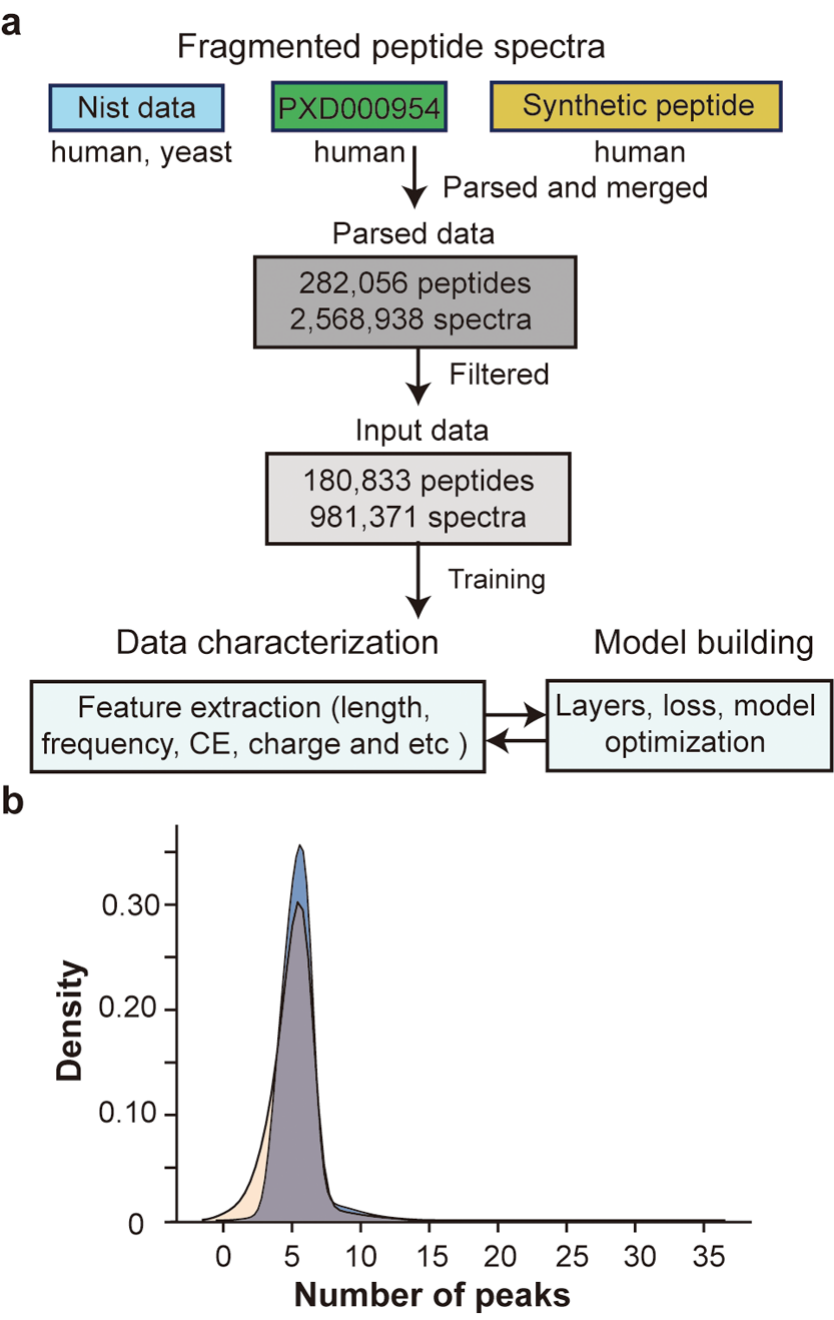
Training data preparation. (a) Schematic diagram of the training data preparation. (b) Density plot showing the distribution of the number of peaks per peptide spectrum before and after filtering the input data. The blue and light-yellow filled graphs indicate the distribution before and after filtering, respectively.

To build a predictive model for peptide fragmentation, we assembled a database from several QTOF data which show high similarity to QqQ-CID. The human and yeast data from the NIST peptide library, data from a study of human proteins by SWATH-MS (PXD000954), and data from laboratory-synthesized peptides were assembled to construct the initial training database.^18,19^ The initial database was parsed to the NIST database format and further parsed to remove redundant peptides. The removal criteria were dependent on the number of identified fragments per peptide, as more y ion peaks per peptide would greatly benefit the training algorithm (Figure 1a). Accordingly, we further removed peptides that had less than three spectra per peptide. The generated database for training consisted of 180,833 unique peptides with an average of 5.427 peaks per peptide (Figure 1b).

### Model Development and Peptide Spectra Characterization Using the Highest Peak

The initial model for peptide spectra prediction was developed with a simple-structured RNN model, with two RNN layers acting as an encoder and decoder. During model development, several editions, such as adding attention layers, testing different dropout probabilities, and changing hidden and batch size, have been performed until the model reached a point where its accuracy reached a plateau (Supporting Information Data S2). Theoretically, bidirectional RNNs should be able to learn the important features of the data and remember their weights in one or some of the nodes. However, in many cases, models improve depending on the type of features and weights that have been appropriately placed in the algorithm. To this end, we attempted to characterize the peptide fragmentation pattern by investigating the affiliation between the peptide and its highest peak (production ion with the most abundance per spectrum) after fragmentation.

**Figure 2:**
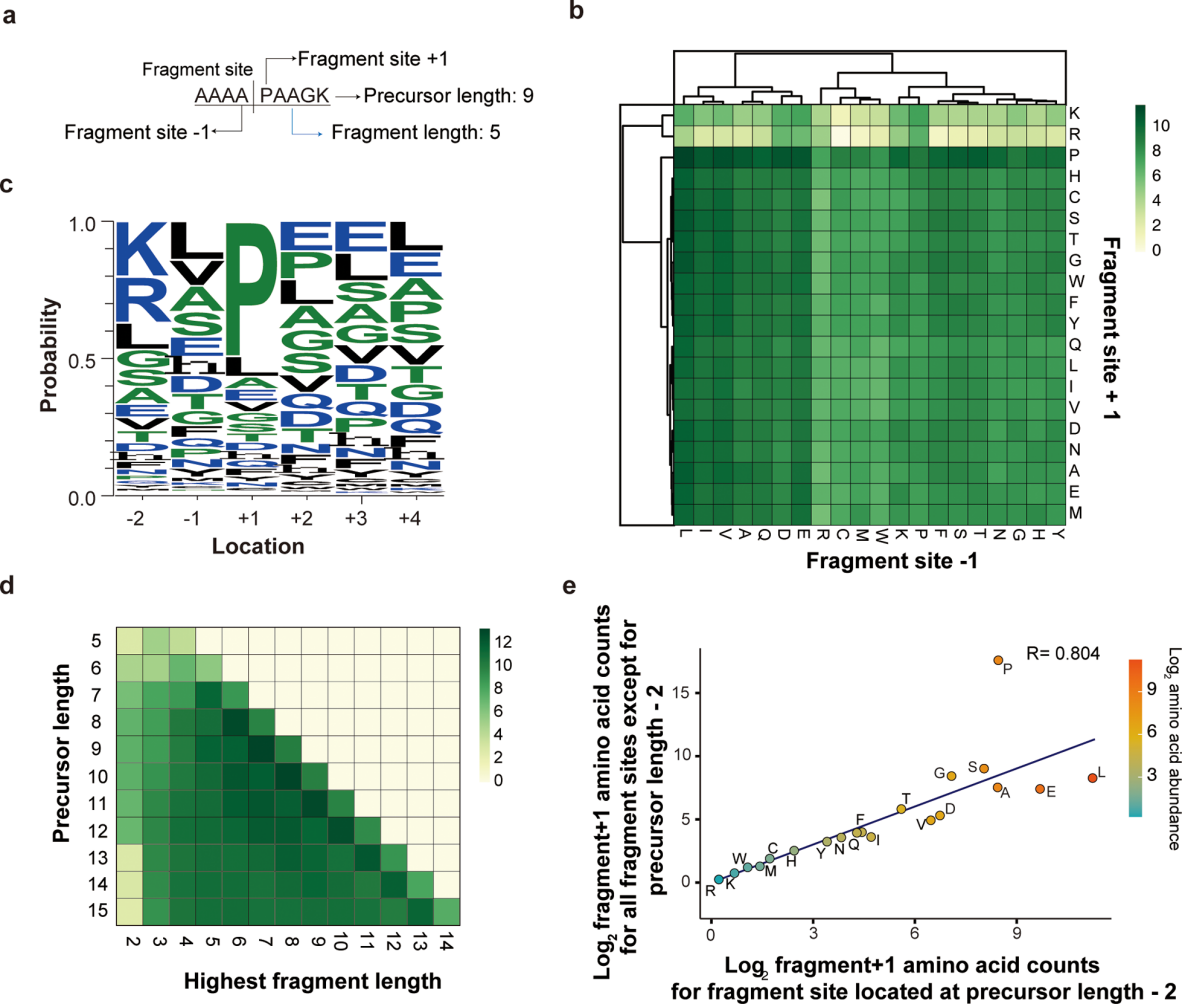
Peptide feature characterization. (a) Schematic diagram of the words describing peptides, peptide fragments, and their location in reference to the fragment with the highest intensity (I.E., y ion with the highest intensity value for the peptide precursor). (b) Heatmap showing the abundance of amino acids for fragment site +1 and fragment site −1 at the vertical and horizontal axes, respectively. (c) Plot of WebLogo showing the probability of amino acid abundance in relation to the fragment site for product ions that retained their charge after fragmentation. (d) Heatmap showing the abundance of the precursor length and highest fragment length at vertical and horizontal axes, respectively. (e) Scatterplot showing the correlation between the amino acid count at fragment +1 sites, for all fragment sites, except for the precursor length −2 and the amino acid count at fragment +1 sites that have the length of precursor length −2.

First, the amino acid sequence patterns before and after the site of fragmentation were investigated (Figure 2a). Heatmap of the amino acid residues show proline residue enrichment at fragment +1 site, regardless of the sequence at the fragment −1 site (Figure 2b). To investigate the effect of proline at the fragment +1 site, more analysis was performed on the peptides that contain proline residues. Proline residues at the +1 site were found to be more abundant for peptides with a high difference between the highest and second highest fragment intensities (Supporting Information Figure S2a,b). Furthermore, proline residues were highly enriched for product ions that retained the same charge state as the precursor ion. More than 50% of the product ions that retained their charge were composed of proline residues (Figure 2c). Although the mechanism behind these results is unclear, the existence of proline residues in a peptide showed sufficient anomalies to be taken as an additional feature to the prediction model.

The fragment −1 site, on the other hand, showed enriched frequency to aliphatic and acidic amino acids. This pattern along with the highly enriched proline residue at the fragment +1 site agrees with previous studies that examined the amino acid sequence of peptide fragmentation.^8^ Moreover, if a single sequence pattern shows such patterns, we suspect that the combination of fragment +1 and fragment +2 sites with fragment −1 and fragment −2 sites would also greatly affect the algorithm. Thus, we implemented a sliding window pattern as one of the key features in the developing algorithm.

Next, we investigated whether the precursor length asserts any effect on the highest fragment length (Figure 2d). Interestingly, we found that the length of the highest fragment was considerably more abundant at precursor length −2 and slightly more abundant at precursor length −4. The length-dependent pattern was especially clear for peptides with precursor lengths between 7 and 12. For longer peptides (>12), the enrichment pattern was weakened, and fragment sites were more evenly distributed at the middle region of the peptide. To check whether any amino acid residues are enriched at the precursor length −2, the abundance of amino acids at the precursor length −2 sites and all other sites were compared (Figure 2e). The correlation graph shows that most amino acid abundances correlate except for proline, which is expected as proline shows an exceptionally high frequency of fragmentation compared to other amino acids. These data indicate that the abundance of amino acids at a given location was similar except for proline, which was enriched indifferentto the precursor length. Thus, the strong influence of the precursor peptide length was added as another feature for the fragment prediction model.

**Figure 3:**
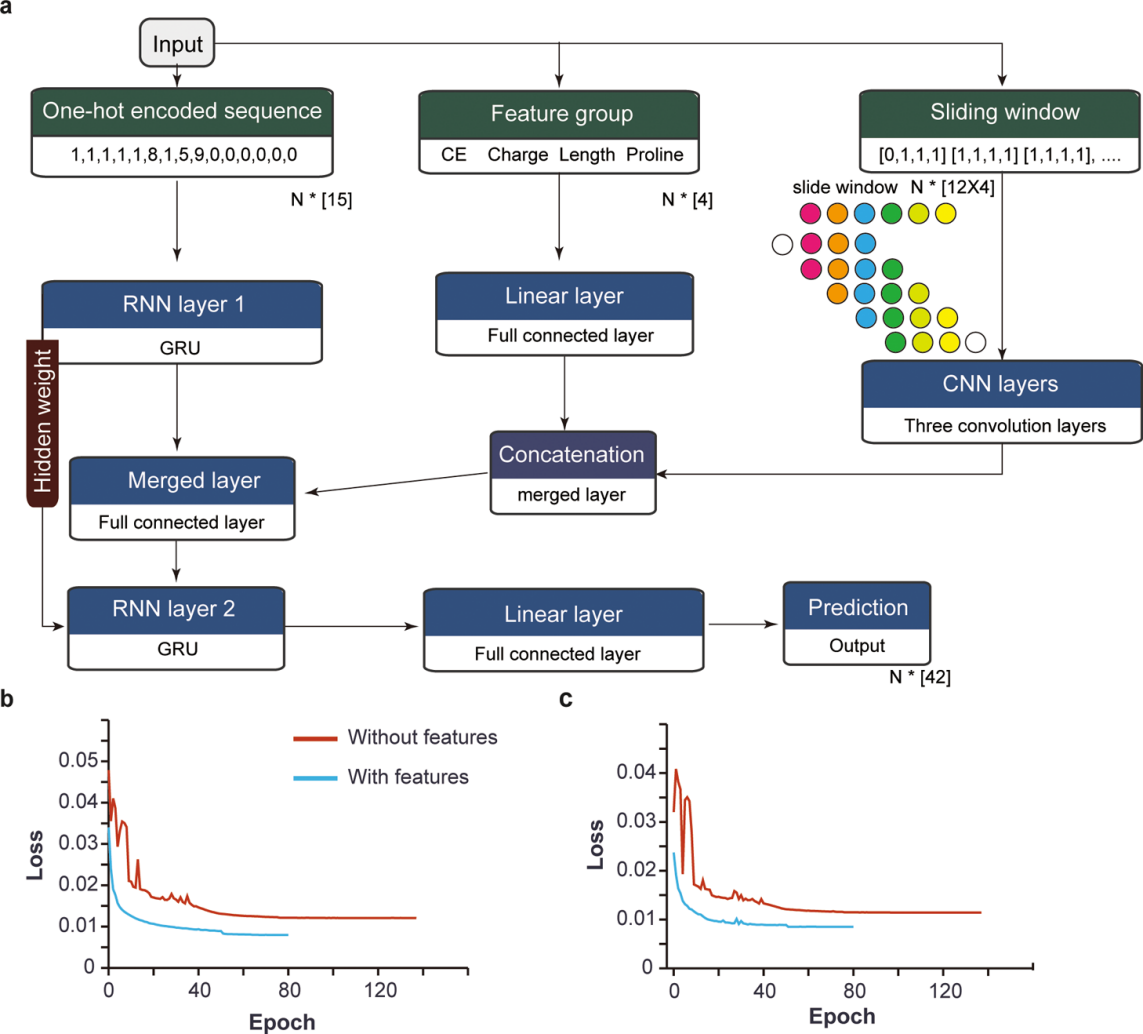
Model structure. (a) Schematic representation of the deep learning model structure. From the input data, the amino acid sequences were one-hot encoded and trained via a bidirectional RNN layer. The information from the feature group was fed to the model by a fully connected layer. The amino acid sliding window information was fed to the CNN layer and represented by the colored circles on the top right side of the figure. The line graph showing the change in loss per epoch with and without features for (b) training data and (c) validation data.

The generated model takes in the peptide sequence, charge, and CE as inputs to calculate the characterized features such as length, proline counts, and sliding windows (Figure 3a). CE, charge, length, and the number of prolines in each peptide were used as feature set 1, where its data flow through a series of linear layers and are concatenated. The feature set 2 is composed of the peptide’s sliding window data, which was shaped linearly at first and then transformed into a two-dimensional layer similar to one-channel image data and trained via CNN (Supporting Information Figure S3). The obtained inputs of peptide sequence and features thus flow through a series of RNNs, CNN, and linear layers to finally predict 42 fragment intensities for a peptide (Supporting Information Table S1). The added features obtained from investigating the highest peak of each peptide enabled better performance of the model (Figure 3b,c). The initial and final loss values for both training and validation datasets were decreased with the representative features, and the number of epochs required for training was reduced.

### Model Evaluation

To evaluate the model, PCC was calculated for each peptide by comparing the predicted spectrum values to the database values. The rat QTOF data from NIST (2013-06-05) was used as the evaluation dataset for 10-fold models (Supporting Information Table S2). NIST rat data were parsed in a similar format to the training data (Supporting Information Data S3). Peptides redundant to the training and validation data were removed, which left 3709 unique peptides with 27,121 spectra after parsing. The average median PCC value obtained from 10-fold cross-validation is 0.944, and the standard deviation of the median PCC value was approximately 0.000129 (Supporting Information Table S2). The low deviation value between the 10-fold models indicates the uniform performance of the trained models, whereas a model with overfitting problem would show high variance from cross-validation. These data indicate that the generated model performs at a similar level of accuracy.

**Figure 4:**
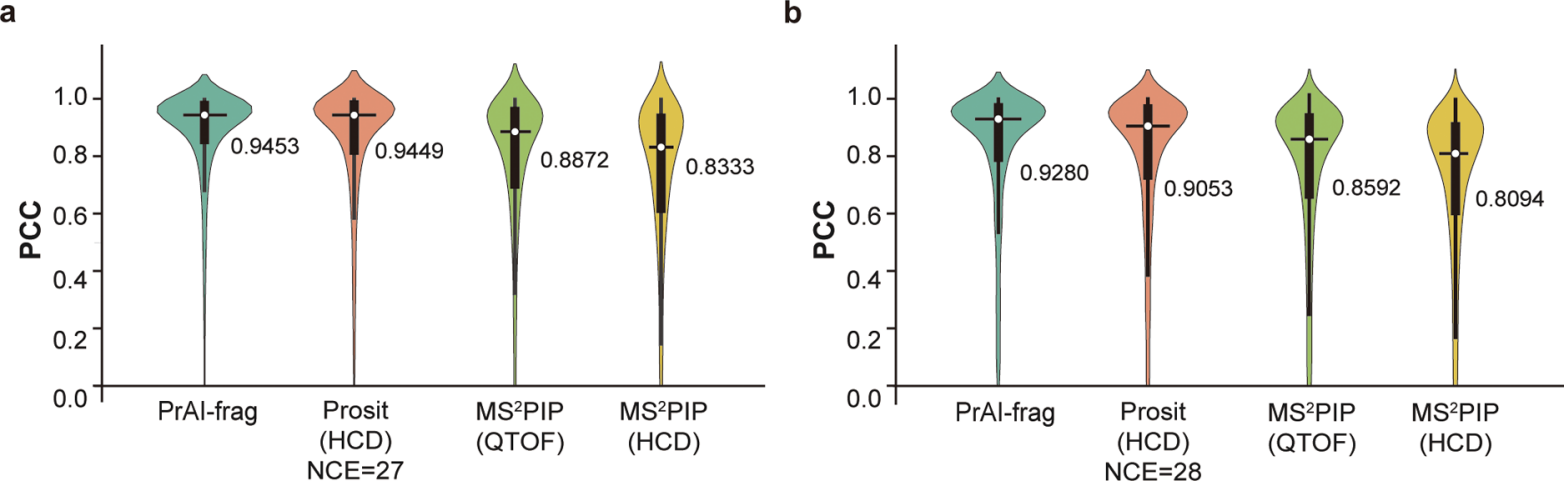
Model comparison. Violin plots showing the PCC of the prediction results from PrAI-frag, Prosit_2020_intensity_HCD (NCE = 27), Prosit_2020_intensity_HCD (NCE = 28), MS^2^PIP QTOF, and MS^2^PIP HCD for the rat QTOF data from the NIST database. (a) PCC calculation performed with “without-zero” data, which uses the input values that only exist in the database. (b) PCC calculation performed with “with-zero” data where zero values are filled in for positions where intensity detection is possible. Only values above zero are shown in the graph.

The final model (hereinafter PrAI-frag) was evaluated further by comparing the PCC distributions calculated from the prediction of Prosit 2020 HCD, MS^2^PIP QTOF, and HCD models to the rat QTOF data (Supporting Information Data S3).^9,11,19,27^ To apply the Prosit HCD model to the evaluation data, we tested all possible normalized CE (NCE) values for CE calibration (Supporting Information Figure S4). The Prosit HCD model with NCE values of 27 and 28 showed the highest correlation to the NIST rat QTOF data for the without-zero and with-zero calculation methods, respectively. The PCC values of approximately 0.945 and 0.905 for the without-zero and with-zero methods, respectively, indicate the Prosit HCD model’s compatibility to the QTOF data (Figure 4a,b). The median PCC of PrAI-frag showed the highest PCC among the compared models of MS^2^PIP and Prosit for both with-zero and without-zero comparisons (Figure 4a,b). While the overall values of PCC decreased for all models by the with-zero method calculation, PrAI-frag showed the least reduction in PCC values (Figure 4b). To further analyze the models’ accuracy, we also calculated the PCC for different peptide precursor charge states. All models showed a slightly increased prediction accuracy for charge 2 and decreased prediction accuracy for charge 3 (Supporting Information Table S3). However, the magnitude of accuracy changes differed for the compared models. PrAI-frag and Prosit models showed 0.1–0.2 decrease in PCC, while the MS^2^PIP model showed a greater decrease in PCC for peptides with charge 3. Nonetheless, these results suggest that PrAI-frag shows robust prediction accuracy.

To assess the effect of the prediction accuracy of different models on the downstream process, we designed two tests. The first test simulated an MRM analysis where a few discriminative transitions, that is, the intense peaks such as the highest peaks need to be selected. To this end, we counted the instances where the predicted highest peak for a peptide spectrum was equal to the highest peak from the database, using the NIST rat data. In agreement to the violin plot, PrAI-frag showed the highest accuracy for the prediction of top three highest intensity peaks (Table 1).

**Table 1:** Prediction Accuracy of PrAI-Frag, MS^2^PIP, and Prosit for High-Intensity Peaks^a^

	highest peak^a^ (%)	top 1 from top 3 (%)	top 2 from top 3 (%)
PrAI-frag	67.835	84.335	68.563
PrAI-frag^b^	68.023	83.770	64.896
Prosit_HCD_27	64.087	81.019	63.629
MS2PIP_HCD	51.766	79.401	54.354
MS2PIP_QTOF	55.055	79.401	57.482

a Highest peak, the number of instances where the predicted highest intensity peak is equal to the highest intensity peak from the database; top 1 from top 3, the predicted highest intensity peak is among the top three highest intensity peaks from the database; top 2 from top 3, the number of instances where the predicted top two highest intensity peaks are among the top three highest intensity peaks from the database.

b Altered version of PrAI-frag with higher weight implemented to higher intensity peaks.

The second test simulated a simplified peptide spectrum match analysis, where peptides with similar precursor ion *m*/*z* (±0.5) that also contains at least three product ions with similar *m*/*z* (±0.5) are used to generate a candidate group of peptides (Supporting Information Data S3). For complex samples, such as serum, the peptides that belong to the same group would be potential noise peaks for one another during the identification process. For the NIST rat data, the grouping resulted in 1822 groups, with 2.548 peptides per group. We next counted the instances in which the model’s predicted peptide spectrum accurately matches the target peptide, against other peptides in the group. Match was performed by selecting the highest PCC and MSE values calculated from comparing all the predicted peptide spectrums and all peptide spectrums in the database within the group. The test showed that PrAI-frag shows the highest match accuracy among the models (Supporting Information Table S4).

For both downstream tests, the accuracy difference between the PrAI-frag and Prosit HCD models (NCE = 27) was in close competition. To confirm the PrAI-frag performance, the first downstream test on the highest peak prediction was performed on two additional QTOF databases (PXD001587 and PXD008651) (Supporting Information Data S3). The highest peak prediction accuracy for the additional database showed that PrAI-frag reproduced the highest prediction accuracy among the compared models (Table S5). It is also noteworthy that the best prediction model for Prosit differed for each database. For PXD001587 and PXD 008651, the Prosit model with NCE values 23 and 25, respectively, showed the best results. Overall, these results suggest that PrAI-frag outperforms other models for high-intensity peak prediction. This is advantageous for applicability to MRM, where fewer and higher intensity peaks are used per peptide.

During model development, we also tested an altered version of PrAI-frag to increase the accuracy on specific target fragments. One example was the prediction of the highest peak (Supporting Information Data S4). By generating a second loss function that calculates the MSE for the fragments with the top three intensities and combining the loss function to the original MSE, the model showed slightly increased accuracy for the highest peak prediction. However, the overall correlation suffered more greatly than that expected and was thus unused in the final model (Table 1).

## Conclusions

Advances in MS/MS technologies and deep learning algorithms have led to the generation of a number of peptide spectrum prediction algorithms. However, to our knowledge, many of the studies used the data obtained from HCD fragmentation, followed by different trapping methods. In this study, we develop a more accurate and QTOF CID-specific peptide fragmentation prediction algorithm. The k-fold cross-validation results showed reproducible training results without overfitting, which also indicated the applicability of the algorithm with additional or different types of data. PrAI-frag was compared with Prosit and MS^2^PIP’s due to their applicability to beam-type fragmentation methods. The comparison of the predicted accuracies of the peptide spectrum and downstream tests indicated that PrAI-frag is highly robust and accurate.

The relationship between the length of the precursor peptide and the fragment length of the highest peak led to unexpected observations. The fragment with the highest intensity was highly enriched at the site of precursor length −2 (Figure 3d). Similar patterns have been reported for doubly-protonated short peptides (5–7 amino acids) fragmented with IT-CID.^28^ The reported protonated oxazolone structure of b_2_ ions indicates that the precursor peptide in the gas phase leads to a structure, which may increase the chance of fragmentation caused by the electrical potential. On the other hand, the fragment with the highest intensity was also enriched at the sites of precursor lengths −4 and −5, for peptides of lengths 8 to 13.^29^ One reason behind the effect of precursor length distribution may be the different protonation distribution to N-term and C-term which may increase the chance of peptide structure formation during acceleration caused by the electric potential difference.^30^ Although it is a speculation, such information applied to deep learning models may improve the accuracy of prediction.

The model building in the present study was designed to map important features from both the peptide fragmentation pattern and the RNN model itself. Unlike the hidden and automatic feature finding from training, feature exploration from the fragment pattern was controllable. We hypothesized that by utilizing peptide fragments with the highest intensity as the representative feature, extra weight would be enforced on the prediction accuracy of peaks with relatively higher intensity. This hypothesis proved correct, which is evident in the comparison of the actual accuracy of prediction of the top three fragments (Table 1). PrAI-frag was able to predict the fragment with the highest intensity for each peptide with higher accuracy compared to MS^2^PIP and Prosit. The accuracy of the highest peak prediction from validation data during training reached over 75%. These data demonstrate the applicability of our algorithm to MRM transition selection. It is also noteworthy that PrAI-frag used 0.98 million spectra for training, while the Prosit 2020 model used approximately 30 million spectra. The substantially smaller training data led to peptide length limitations of 15 amino acids. Nevertheless, we expect our model to continuously improve with more data and tests, which would be updated via the Web.

## Supporting Information Available

CE calculation; detailed training model structure; model evaluation; description on the alternative model; DOME machine learning table for PrAI-frag; median PCC values calculated from K-fold cross-validation; median PCC values of models for precursor charge state; simplified peptide spectrum match comparison between models; high-intensity peak prediction of models on additional data; different PCC calculation methods; Weblogo diagram of enriched amino acids; detailed schematics of the training model structure; and Prosit HCD model CE calibration (PDF)
