# Supplementary material for: Accurate Prediction of y Ions in Beam-Type Collision-Induced Dissociation Using Deep Learning
Source: Anal Chem. 2022 May 24;94(22):7752–8. doi: 10.1021/acs.analchem.1c03184 (PMC9178553; doi:10.1021/acs.analchem.1c03184)
Supplement: Supplementary file 1 — ac1c03184_si_001.pdf [file ac1c03184_si_001.pdf]

Supplementary information

Accurate prediction of y-ions in beam-type CID using deep learning

HyeonSeok Shin*, Youngmin Park, Kyunggeun Ahn, Sungsoo Kim

Bio Convergence Research Institute, Bertis inc., Heungdeok 1-ro, Giheung-gu, Yongin-si, Gyeonggi-do,16954, Republic of Korea

Table of contents

Supplementary Data

S-1. Collision energy calculation ……………………………………………………………………S2

S-2. Detailed training model structure ……………………………………………………………S3

S-3. Model evaluation ……………………………………………………………………………S5

S-4. Alternative model description …………………………………………………………………S7

Supplementary tables

Supplementary Table S1. The DOME machine learning table for PrAI-frag ……………………S8

Supplementary Table S2. Median PCC values calculated from K-fold cross validation ………S10

Supplementary Table S3. Median PCC values of models for precursor charge state ……………S11

Supplementary Table S4. Simplified peptide spectrum match comparison between models ...S12

Supplementary Table S5. High intensity peak prediction of models on additional data ………S13

Supplementary Figures

Supplementary Figure S1. Different PCC calculation methods. …………………………………S14

Supplementary Figure S2. Weblogo diagram of enriched amino acids. …………………………S15

Supplementary Figure S3. Detail schematics of the training model structure. ………………… S16

Supplementary Figure S4. Prosit HCD model CE calibration ………………………………… S17

Supplementary Data S1 Collision energy calculation

All applications and models that require collision energy (CE) calculations, training data, evaluation data, and inference missing value fill-in, was calculated using equation S1. The CE value for PrAI-frag was calculated using NCE=29. The *m/z* is the mass/charge value, NCE is a given number for and the charge factor is 0.9 for charge 2 and 0.85 for charge 3. The charge and *m/z* value of each peptide were calculated from python library Pyteomics.

Equation S1. $\mathbf{CE} = \mathrm{NCE}\ \mathrm{X}\ \frac{\mathbf{m/z}}{\mathbf{500}}\ \mathrm{X}\ (\mathrm{charge\ factor})$

Supplementary Data S2 Detailed training model structure.

Inputs. The training model takes in six inputs which were one-hot encoded peptides' amino acid sequence, collision energy (CE), charge, length, the number of proline residue and sliding window of 4-mers. The six inputs were either calculated or converted from peptide amino acid sequences and this information was fed onto the model in three different types of layers. The recurrent neural network (RNN) layer takes in the embedded one-hot encoded peptides' amino acid sequence to learn sequential information from amino acid sequences. The fully connected layer takes in four inputs, CE, charge, length, and the number of proline residues which will here on be called feature_group1. The information in feature_group1 was fed into the model via a single full connected layer as the sequential information was not crucial. The convolutional neural network layer takes in the sliding window of 4-mers. The sliding window of 4-mers contained information on sequentially serial 4-mers of each peptide. For example, a peptide with the sequence of VAGAAVAK is transformed to VAGA, AGAA, GAAV, AAVA, AVAK after sliding window. The sliding window for VAGAAVAK produce 5 sliding windows of 4-mers which is equivalent to peptide length – 3. For a peptide with length of 15, 12 sliding windows of 4-mers can be generated. To feed this information into the CNN layers, the sliding window was designed to be represented in a 12 by 4 shaped two-dimensional (2D) matrix. This shape was equivalent to a single channel image data with 12 X 4 pixels: thus, enabling CNN implementation.

Model architecture. The model is structured to take in three different types of data to different layers of RNN, FCN and CNN which is then combined to be decode on a second RNN layer. The one-hot encoded sequence were fed onto bidirectional gated recurrent units (GRU) layer with hidden size of 128 where the hidden states were saved and

forwarded to the second GRU layer described later. The GRU layer output was subsequently forwarded to dropout layer (p=0.4), leaky rectified linear unit ReLu with 0.3 gradient and to attention mechanism layer with dropout (p=0.1). The output after dropout was forwarded to a feature size of 256 which is later multiplied to the output matrix concatenated from feature 1 FCN layer and sliding window CNN layers.

The sliding window of 4-mers were inputted with 12 X 4 matrix is forwarded to 2D convolution layer with kernel size of 1 X 4, stride of 1 and ReLu function to a 12 X 1 matrix with 48 channels. The output was subsequently forwarded to the second 2D convolution layer with kernel size of 2 X 1, stride of 1 and leaky ReLu function with 0.3 gradient to a 11 X 1 matrix with 128 channels. The output was forwarded to the third convolution layer with kernel size of 11 X 1 and stride of 1 and dropout (p=0.4) to a 1 X 1 matrix with 14 channels. The output from 2D convolution layers was flattened to feature size of 224. The output of the sliding windows of 4-mers are concatenated to the output of a single FCN layer from feature 1 which forwards 4 features to feature size of 32. The concatenated output from sliding window of 4-mers and feature_group1 results in a feature size of 256 which was multiplied to the output from the first GRU layer also with a feature size of 256 and fed to the second GRU layer.

The second GRU layer takes in the multiplied feature and the hidden states from the first GRU layer. The output from the second GRU layer was forwarded to dropout layer (p=0.4), second attention mechanism layer with leaky ReLu (0.3 gradient). The output with features size of 256 are subsequently reduced to 128 and finally to 42 as the final output.

Supplementary Data S3 Model evaluation

Model parameters. For model comparison, rat QTOF data obtained from NIST databases (2013-06-05), *Escherichia Coli* QTOF data obtained from PRIDE (PXD001587) and *Mus Musculus* QTOF data obtained from PRIDE (PXD008651) were used as the evaluation database which were parsed in similar format of the training database. Peptides with less than three peaks and peptides of length higher than 15 were removed. Redundant peptides with the training database were removed which left 3,709 tryptic peptides for evaluation. Modifications, such as carbamidomethylation on cysteine was ignored. Evaluation was performed using the NIST rat data unless mentioned otherwise. The compared models were Prosit_2020_intensity_hcd, MS^2PIP_QTOF and MS^2PIP_HCD. For the Prosit_2020_intensity_hcd model, 22 NCE combination from NCE 18 to NCE39 have been tested (**Supplementary Fig. S3**). The CE value of each peptide that were required as input for Prosit, were calculated using equation S1 (**Supplementary Data. S1**). The MS^2PIP was tested without modification option for TripleTOF 5600+ model and HCD model.

Model comparison for simplified peptide spectrum match analysis. To simulate a simplified peptide spectrum match analysis, we first grouped peptides from the NIST rat data by *m/z* similarity. Peptides with similar *m/z* values (± 0.5) for the precursor ion was grouped which resulted in 3,658 groups, where each group had an average of 12.154 peptides per group. Among the grouped peptide, we searched for peptides that contains at least three product ions in similar *m/z* values (± 0.5) which reduced the number of groups to 1,822 groups with 2.548 peptides per group. The grouping was performed to simulate an MRM analysis without standard (heavy peptides) where multi-peaks were observed for the targeted *m/z* transitions. For every group, there exists a “target peptide”

which is the actual peptide we want to deduce. The other peptides that were within *m/z* ± 0.5 range to the target peptide were noises that cannot be easily differentiated. Each model than predicts the fragmentation spectrum of every peptide in the group which was compared to the actual target peptide fragmentation spectrum. This was repeated until every peptide in the group has been the "target peptide". The similarity between all peptides in the group against the target peptide was calculated by PCC and mean squared error (MSE) for all spectrum or for the highest 3 intensity peaks. To avoid bias occurring from MSE calculation, all model's predicted maximum intensity value for each peptide was normalized to 1, by dividing with the maximum value. The accuracy of each model was calculated by counting the number instance where maximum scoring peptide was equivalent to the target peptide.

Supplementary Data S4 Alternative model description.

The altered version of the PrAI-frag shares the same structure of the PrAI-frag. The loss function, however, was altered to impose greater weight on the accuracy of the highest intensity. The mean squared error (MSE) function used in PrAI-frag estimates the error rate of the total output which would be the difference between 42 intensity per peptides.

Supplementary equation S2. MSE loss function

$$Loss = \frac{1}{n}\sum_{i=1}^{n}(y_i - t_i)^2$$

The altered loss function was composed of two MSE loss function, where the original MSE value of the total intensity is added to the MSE value of only the top 3 highest intensity peaks are calculated. The a, b, and c in the equations each represents the top1, top2 and top3 highest intensity fragments. The m and n in the equation is the varied weight given to each loss function, where 0.3 and 0.7 was used for m and n, respectively.

Supplementary equation S3. Altered loss function

$$Loss = m \times \frac{1}{n}\sum_{i=1}^{n}(y_i - t_i)^2 + n \times \frac{1}{3}\left((y_a + t_a)^2 + (y_b + t_b)^2 + (y_c + t_c)^2\right)$$

Supplementary Table S1. The DOME machine learning table for PrAI-frag.

**DOME**	Version	1.0
**Data**	Provenance	Total used peptide spectrum data – 211,026 peptides, 1,219,173 spectrums (Total data used including training, validation and test (evaluation))<br>NIST : collision cell library quadrupole time-of-flight (QTOF) of human data (2012-04-20), rat data(2013-06-05) and yeast data (2012-04-17)<br>SWATH-MS : Proteomics Identification Database, PRIDE Project: The Pan-Human Library: (PXD000954)<br>PRIDE Project: DIA-Umpire: (PXD001587)<br>PRIDE Project: SWATH mass spectrometry as a tool for quantitative profiling of the matrisome (PXD008651)<br>647 laboratory synthesized peptides
	Dataset splits	Training data : 162,750 peptides<br>Validation data : 18,083 peptides<br>Train + Validation data: 180,833 peptides, 981,371 spectrums<br>Training and validation data consists of PRIDE Project (PXD00954), Nist human data, Nist yeast data and 647 laboratory peptides.<br>Test dataset 1: 3,709 peptides, 27,121 spectrums (NIST rat data)<br>Test dataset 2: 3,456 peptides, 16,530 spectrums (PRIDE PXD 001587)<br>Test dataset 3: 23,028 peptides, 194,151 spectrums (PRIDE PXD008651)
	Redundancy between data splits	No, training sets and test sets are independent, redundant peptides were removed.
	Availability of data	Yes, NIST https://chemdata.nist.gov/dokuwiki/doku.php?id=peptidew:cdownload<br>SWATH-MS<br>https://www.ebi.ac.uk/pride/archive/projects/PXD000954<br>https://www.ebi.ac.uk/pride/archive/projects/PXD001587<br>https://www.ebi.ac.uk/pride/archive/projects/PXD008651
**Optimization**	Algorithm	Neural network<br>- GRU<br>- Attention<br>- Convolution
	Meta-predictions	No
	Data encoding	Peptide sequence<br>- One-hot encoding<br>Peptide feature<br>- Calculation peptide collision energy, length of sequence and number of proline<br>- Calculated sliding window on one-hot encoded sequence

	Parameters	Total parameters : 791,622<br>Trainable parameters : 791,622
	Features	- One-hot encoded sequence<br>- List of peptide charge, peptide collision energy, length of sequence, number of proline<br>- Sliding window on one-hot encoded sequence
	Fitting	Not applicable.
	Regularization	No
	Availability of configuration	https://github.com/bertis-prai/PrAI-frag
**Model**	Interpretability	Black box, as correlation between input and output is masked. No attempt was made to make the model transparent.
	Output	Regression - Peptide fragment intensity
	Execution time	1,300 sec ~ 1,600 sec per fold of training dataset
	Availability of software	Website and github.<br>http://www.prai.co.kr/
**Evaluation**	Evaluation method	- 10 fold cross-validation<br>- Test model with independent dataset (NIST rat data)
	Performance measures	- Pearson correlation coefficient<br>- Accuracy of top 3 fragment intensity
	Comparison	Prosit<br>- Prosit_2020_intensity_hcd (NCE18 to 39)<br>MS2PIP<br>- HCD model<br>- TripleTOF 5600+ model
	Confidence	Not calculated
	Availability of evaluation	No.

Supplementary Table S2. Median PCC values calculated from K-fold cross validation

K-fold	PCC [α]
Fold0	0.944626
Fold1	0.94293
Fold2	0.945369
Fold3	0.944754
Fold4	0.944109
Fold5	0.943897
Fold6	0.942483
Fold7	0.946704
Fold8	0.943205
Fold9	0.942437

[α] The PCC values were calculated using the NIST rat data for 10 models that has been trained for different folds.

Supplementary Table S3. Median PCC values of models for precursor charge state

		Charge2	Charge3
Number of peptides		3561	143
with zero	PrAI-frag	0.9323	0.8265
	Prosit-HCD (NCE=28)	0.9083	0.7767
	MS2PIP_QTOF	0.8651	0.4661
	MS2PIP_HCD	0.8159	0.4176
without zero	PrAI-frag	0.9473	0.8582
	Prosit-HCD (NCE=27)	0.9459	0.9014
	MS2PIP_QTOF	0.8919	0.3931
	MS2PIP_HCD	0.8391	0.3524

Supplementary Table S4. Simplified peptide spectrum match comparison between models

Method [α]	PrAI-frag (%)	Prosit NCE 27(%)	MS^2PIP QTOF (%)	MS^2PIP HCD (%)
PCC	85.950	85.950	83.205	79.748
Top3_PCC	68.990	67.673	60.538	60.538
MSE	86.883	86.059	61.196	61.306
Top3_MSE	77.991	76.948	70.252	68.222

[α] The method indicates the calculation method used to compare the similarity between predicted peptide spectrum for each peptide spectrum in a group. PCC and MSE were calculated using the without-zero method. Top3 PCC and top3 MSE indicates that calculation was performed for the top three intensity peaks in the database.

Supplementary Table S5. High intensity peak prediction of models on additional data

	Highest peak	Top1 from top 3	Top2 from top 3
PXD001587			
PrAI-frag	65.60%	83.54%	63.43%
Prosit_HCD_27	62.47%	77.26%	57.03%
Prosit_HCD_23	64.12%	78.18%	58.10%
MS^2PIP_HCD	56.68%	82.73%	58.83%
MS^2PIP_QTOF	55.73%	85.42%	60.42%
PXD008651			
PrAI-frag	49.44%	70.39%	47.34%
Prosit_HCD_27	44.93%	65.17%	39.70%
Prosit_HCD_25	45.12%	65.59%	39.74%
MS^2PIP_HCD	39.32%	61.35%	33.21%
MS^2PIP_QTOF	42.94%	63.70%	37.10%

Supplementary Figure S1

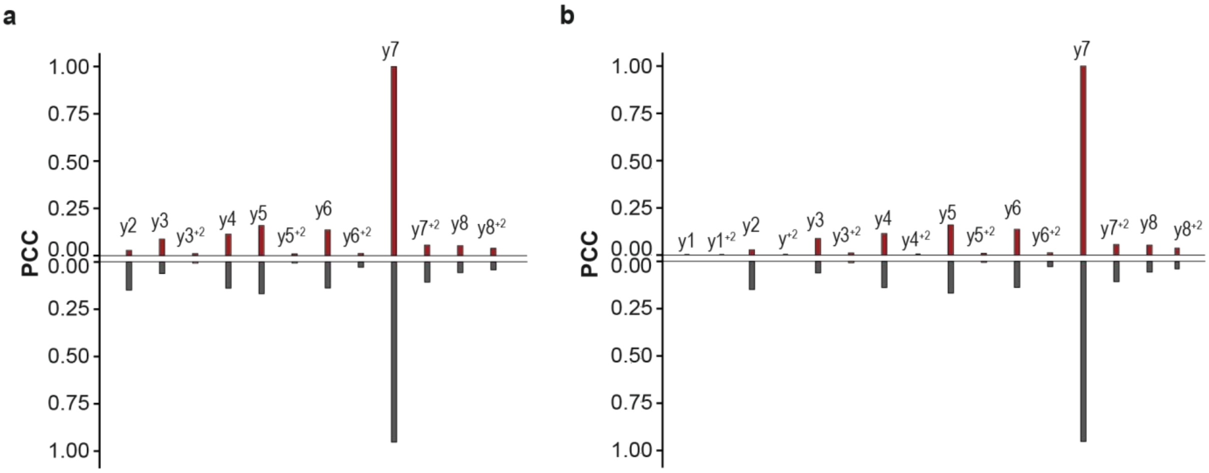

Supplementary Figure S1. Different PCC calculation methods. A line plot showing the intensity value of VVSYQLSSR (a) without zero and (b) with zero values. The predicted values are shown in red and the value from the database are shown in grey. The "without zero" method calculates PCC by comparing the prediction to only existing values from the database. The "with zero" method calculates PCC by comparing the prediction to zero values filled into positions where intensity detection was possible but were not detected from the original database, such as $Y4^{+2}$, $Y2^{+2}$, $Y1^{+2}$ and Y1.

Supplementary Figure S2

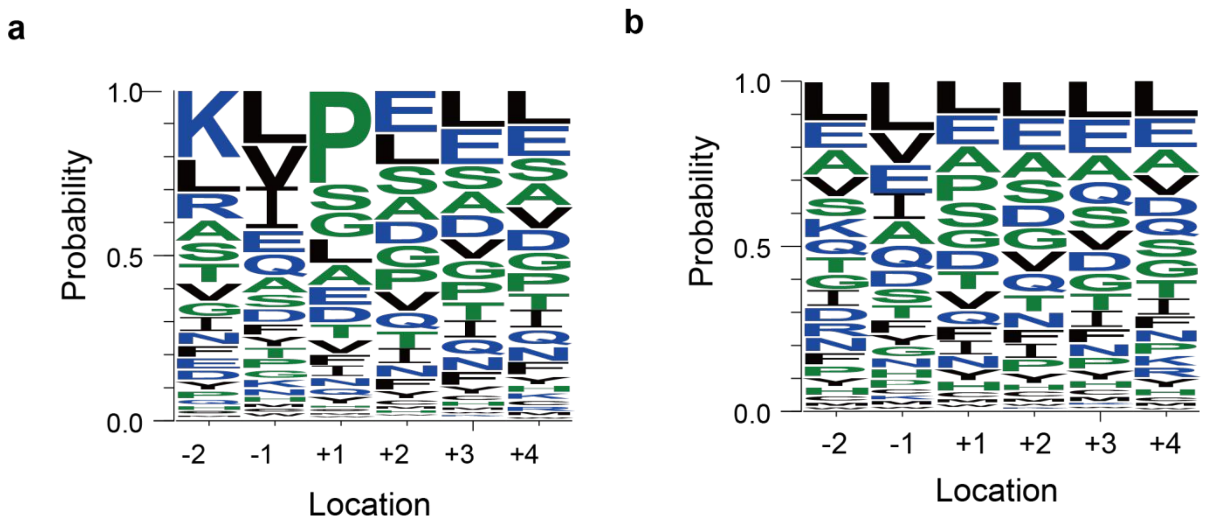

Supplementary Figure S2. Weblogo diagram of enriched amino acids. Weblogo diagram showing the amino acid probability for peptides with difference between the highest and second highest fragment intensity for the (a) highest 10% and (b) lowest 10%. The location on the X-axis indicates the fragmentation position for the highest fragment intensity.

Supplementary Figure S3

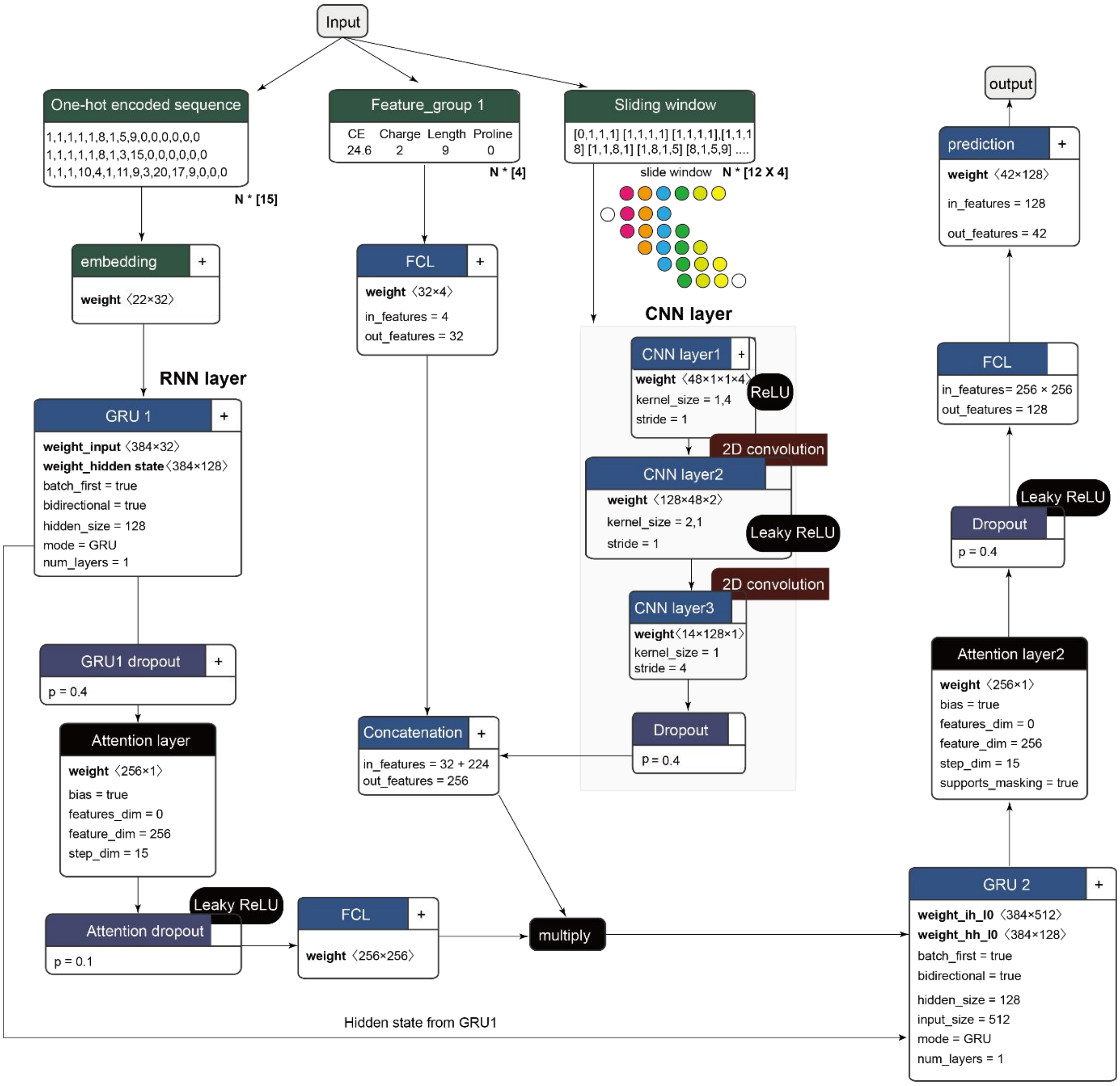

Supplementary Figure S3. Detail schematics of the training model structure. Letter N, under the input shape represents the batch size. Letter P, under dropout represents probability. Abbreviations are as follows; collision energy, CE; full connected layer, FCL; gated recurrent unit. GRU; leaky rectified linear unit, ReLu.

Supplementary Figure S4

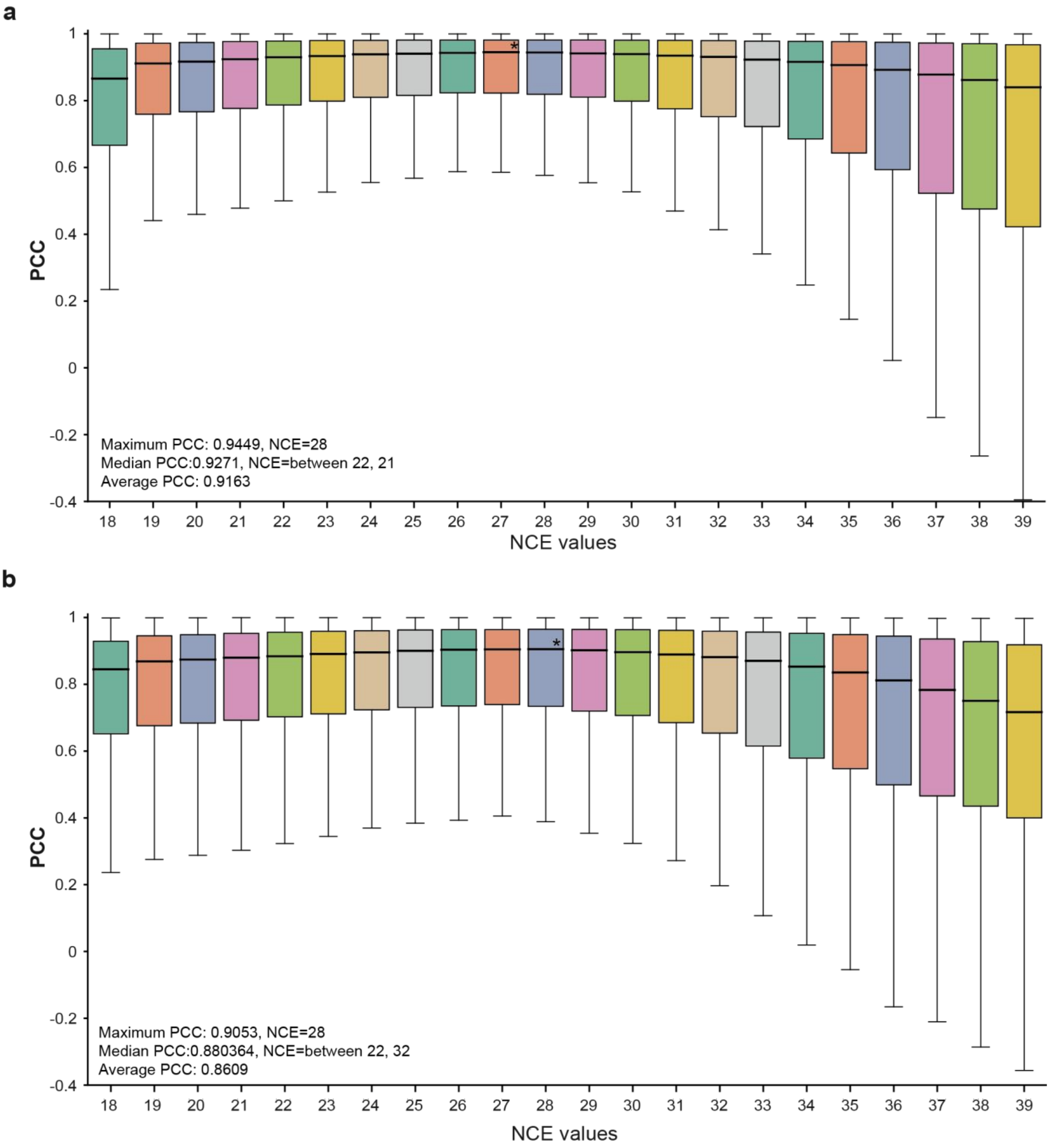

Supplementary Figure S4. Prosit HCD model CE calibration Box plot showing the PCC distribution of the predicted results from Prosit_2020_intensity_HCD for all possible NCE values from 18 to 39 for the rat QTOF data from Nist database. The PCC calculation was performed with (a) only the input values that exist in the database and (b) input values with zero filled in for peaks with absent intensity. The asterisk on top of the median line indicate the box plot with the highest median value among the NCE models.
